# A cross-sectional household cluster serosurvey of hepatitis C virus antibodies in an urban slum of Cairo, Egypt in 2004

**DOI:** 10.1186/s40794-015-0012-7

**Published:** 2015-10-01

**Authors:** Isabelle A. Nakhla, John W. Sanders, Emad W. Mohareb, Sahar Samy, Michael T. Cosby, Manal M. Mostafa, Mark S. Riddle, Robert W. Frenck

**Affiliations:** 1 Naval Medical Research Unit #3, PSC 452, Box 5000, FPO AE 09835-9998 Cairo, Egypt; 2 Wake Forest University School of Medicine, Medical Center Blvd., Winston-Salem, NC 27157 USA; 3 Egyptian Ministry of Health, Cairo, Egypt; 4 Cincinnati Children’s Hospital Medical Center, Cincinnati, USA

**Keywords:** Hepatitis C virus, Prevalence, Risk factors, Cluster survey, Garbage collectors, Occupational exposure, Egypt, Slum, Needles, Tatoo

## Abstract

**Background:**

Hepatitis C Virus (HCV) continues to be a burden to the Egyptian population and its economy. Despite all efforts, the prevalence of infection continues to be one of the highest in the world. The high national prevalence has been attributed to unintentional, nosocomial spread during an anti-schistosomiasis campaign conducted in the 1970’s, but that does not fully explain the persistent infection rates. Work is needed to further clarify risk associations. A serosurvey was performed in Manshiet Nasser, a slum in Cairo sometimes referred to as Mokattem Hills where a primary occupation is garbage collection and sorting, to characterize potential risk factors for infection.

**Methods:**

Following a detailed mapping and census of the area, a cluster sampling was performed and demographic and risk behavior data and a blood sample were collected from subjects older than 6 months. Blood samples were tested using 4^th^ generation anti-HCV EIA kit.

**Results:**

The slum was estimated to house 45,000 residents. Blood samples were obtained from 2169 subjects. The age adjusted anti-HCV seroprevalence was 9.1 %. Participants with HCV antibodies were more likely to be male, heads of households, subjects without formal education, and those with a lower standard of living. After adjustment of all prevalence ratios (aPR) for age, the following risk factors were significantly associated with higher risk of HCV infection: Garbage collection (aPR 1.5), history of blood transfusion (aPR 2.0), tattooing (aPR 1.4), history of schistosomiasis (aPR 1.5), and use of traditional latrines (aPR 2.0) or pits for sanitation (aPR 1.3). The results of the multivariate analysis indicated that age (*p* < 0.01), history of schistosomiasis (*p* < 0.05), garbage sorting (*p* = 0.05), blood transfusions (<0.001), and the use of traditional latrines for sanitation (*p* < 0.01) were significantly associated with infection.

**Conclusion:**

While HCV prevalence among those over 30 could be attributed to anti-schistosomiasis injections, the prevalence in younger age indicates ongoing transmission. Although specific behavioral risks were not identified, HCV infection appears to be an occupational hazard of garbage collection and sorting in this environment. Given the large reservoir of HCV infection in the population, further effort needs to be made to identify and mitigate new infections.

## Background

Hepatitis C virus (HCV) infection is a well-recognized health problem throughout the world and particularly in Egypt. In contrast to an HCV seroprevalence of 1–2 % in the United States and Europe and 0.1 to 5 % in the Middle East, prevalence in Egypt ranges from 11 to 14 % with the highest rates along the Nile River delta [1–9]. The Egypt Demographic Health Survey 2008 (DHS) showed a total prevalence of anti-HCV antibodies of 14.7 % and detectable HCV RNA in 9.8 %. Prevalence was higher in men and rural governorates [10]. It has been suggested that the high prevalence of HCV in Egypt is related to contaminated needles used to administer tartar emetic as part of a campaign to eradicate schistosomiasis in the 1970’s [11, 12]. However, this cannot explain transmission among people born since 1980s, particularly in Cairo and Alexandria where large campaigns against schistosomiasis were not conducted. The epidemiology of HCV from the United States suggests that blood transfusions and intravenous drug use are major modes of transmission, but in Egypt, other modes of transmission such as sexual, intra-familial, and mother to child transmission, have been suggested as well [1, 13].

The Egyptian government recently announced a new strategic plan to address the HCV epidemic [14]. While a deal with the pharmaceutical company, Gilead, to provide their revolutionary new treatment at an affordable cost has received the most attention, the strategy also includes a plan to establish a national system to identify transmission trends and adapt responses as needed. A sentinel surveillance system was started in 2001 in five hospitals and identified ongoing transmission [15]. Unsafe medical injections, primarily through reuse of disposable syringes, was identified as a major risk factor [16], and hence much of the focus of the Egyptian government is to improve infection control practices in the health care system [14]. There is also significant interest in the role of vertical transmission [17]. However, other risk factors such as occupational exposures may be important as well. In order to better evaluate the epidemiology of ongoing HCV transmission in Egypt, we have re-evaluated data from a previously performed cross-sectional survey designed to elucidate the risk factors and prevalence of HCV among a well-defined urban population of high-risk behavior garbage collectors in Cairo.

The study was conducted from 2003 to 2004 in a densely populated urban slum in Eastern Cairo known as Manshiet Nasser, sometimes also referred to as Mokattem Hills. The most common source of revenue for residents of the slum involves garbage collection so it has euphemistically been referred to as “garbage city.” Traditionally, the men and boys have collected garbage from throughout the city, often including medical waste, bringing it back to the slum where the community sorts and recycles it, potentially exposing them to a wide array of infectious agents. It has attracted immigrants from other parts of the country and approximately one quarter of the families moved to the settlement after 1981 [18].

## Methods

The community was mapped using high resolution diagrams to include details of each building. Each of the buildings and each of the units within the buildings was given a discriminate number. From the 2,258 buildings in the community, 15 were randomly selected and censused. This pilot survey found there to be an average of four family units per building and five people per family unit, resulting in an estimated population of the community of 45,000 persons.

Previous studies have estimated the prevalence of HCV in Cairo to be 8 % [19]. Using cluster-sampling sample size calculation with a 99 % confidence interval (CI) and a 2 % margin of error, it was projected that 3136 subjects would need to be enrolled to adequately assess risk associations. Assuming a 70 % participation rate, 4500 people would likely need to be approached to reach the targeted enrollment. Based on the pilot study and sample size estimate, every tenth building was selected to be included in the study and considered as one cluster. All households living in each building were visited and asked to participate in the study. If the household head refused to participate in the study, the next household within the building, as numbered during the mapping of the area, was approached. If all the families in the building refused to participate, the next adjacent building, as numbered during the mapping of the area, was approached for enrollment.

After verbal consent was provided, a socio-epidemiologic survey was performed with a detailed census of the household unit to include demographic characteristics of each member such as gender, age, education level, and occupation. Census information was entered into a computerized database allowing generation of a list of all members of each household that participated in the census and demographic questionnaire. These households were re-visited and each household member over 6 months of age was asked to donate a single sample of blood to be used for detection of antibodies to HCV (see below for methods). Subjects willing to participate in the serosurvey were also administered a structured clinical close-ended questionnaire. Information regarding history of an illness consistent with hepatitis, occupation, and presence of factors suspected of being associated with a higher risk of acquiring HCV were collected. Questionnaires were labeled with a personal identification number (PIN) to protect the confidentiality of the subject yet allow linkage to the blood samples.

A single 3 mL sample of blood was collected in a serum separator blood collection tube labeled with the subject’s Personal Identification Number (PIN). After collection, the samples were allowed to stand at room temperature for 30 min to allow clot formation and then transferred to a cooler until transported to the Naval Medical Research Unit #3 (NAMRU-3) lab at the end of the day. The lab was located approximately 20 min from the study site. At NAMRU-3, the serum was separated and frozen at −70 °C pending testing.

Detection of HCV antibodies was performed using a 4^th^ generation anti-HCV EIA kit (Murex, London UK) as per the manufacturer’s instructions. Positive and negative control wells were performed with each testing plate. For the test to be considered valid the mean negative control absorbance value had to be < 0.25 and the mean positive control absorbance had to be > 0.8 above the mean absorbance value of the negative control. Utilizing the manufacturer’s kit instructions, a cutoff value was determined by adding 0.6 to the mean negative control absorbance results. The sample was considered reactive if its reading was higher than the cut off value. To check for false positive test results, reactive samples were re-tested in duplicate and those samples that were again above the cut off value in at least one of the two re-tested wells were presumed to have antibodies to HCV antigens. Samples that were non-reactive in both wells during the re-test were considered non-reactive.

Study subjects were provided a copy of their lab results. Those found to be anti-HCV positive were counseled regarding the infection as well as methods to prevent spread. Infected subjects also were offered liver function testing accompanied by coordinated referral for further testing (PCR) and management in Ministry of Health facilities where treatment would be offered at minimal expense.

All data was double entered into a Microsoft Access database and checked for consistency. Data analysis was performed using SAS v8.2 (SAS Institute, Carey, NC) and Stata v8 (Stata Corps, College Station, TX). Proportion of respondents and non-respondents were statistically compared using chi-square (Fisher’s exact test when the data were sparsely distributed). For continuous variables difference between groups will be analyzed using *t*-test (Mann-Whitney when parametric assumptions were not met).

Stratum-specific age and gender seroprevalence was determined for the study population. In addition, the census results for the study population were used to estimate age- and gender-adjusted population anti-HCV seroprevalence for the population of the slum. Crude and adjusted prevalence ratios (PR) were calculated to assess the association between risk factors and anti-HCV seropositivity. To estimate the adjusted PR, logistic regression was performed using the generalized estimating equation [20] which enabled an adjustment for the correlation between respondents living within the same family and age effect [21, 22]. After examining the effect of age on anti-HCV seroprevalence, age was stratified into three categories (0–12 years, 13–30 years, and 31 years and greater). Each risk factor and age category was then entered into an equation as independent variables with the anti-HCV positivity used as the dependent variable to check the effect of each risk factor alone. To arrange the risk factors according to their importance, all risk factors with significant effect on infection were entered into one equation with age and anti-HCV status.

Although the entire community was considered to be economically depressed, questions were included in the household questionnaire intended to evaluate the family’s standard of living. These questions included such items as home ownership, availability of some expensive and durable items such as indoor plumbing, a radio, a washing machine, an automobile, a television, etc. Positive answers to these questions were assigned one point and socio-economic status was expressed in scores based off the sums of these questions. Respondents in the lower quartile were considered to be poor relative to their community.

The study was reviewed and conducted as per ethical standards of the Institutional Review Board of the U.S. Naval Medical Research Unit #3 (Cairo, Egypt) and in keeping with the guidelines of the US Department of Defense, which comply with the Helsinki Declaration of 1975.

## Results

### Population survey and demographics

**Fig. 1:**
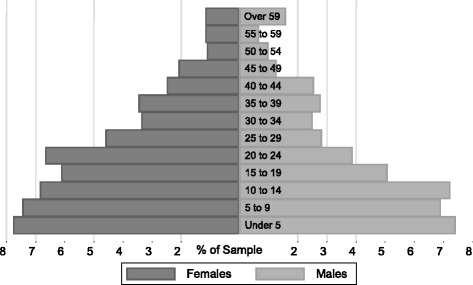
Census Demographic Tree. Age and gender distribution of population assessed by the census of Manshiet Nasser, 2003

Every tenth building was surveyed, and there were no refusals. The sample included 760 families with 3757 subjects residing in 248 buildings. See Fig. 1 for age and gender distribution. The mean age of the surveyed population was 23 years (range 6 months to 90 years, SD 16.1). Fifty-one and one half percent of the subjects were male, 28.1 % did not receive any formal education, and 11.4 % were working in garbage collection, sorting and/or recycling. Of the total sample, 20.4 % came originally from a rural governorate, while the remainder represented individuals from urban governorates (23.0 %) or Manshiet Nasser natives (56.6 %).

### Serosurvey demographics

From 3757 subjects included in the census survey and asked to enroll in the study, 2180 (58 %) respondents presented to the local area medical clinic for blood sampling and completion of the clinical questionnaire. For technical reasons, blood samples could be obtained from only 2169 (99.5 %). There were several differences among the group that contributed a blood sample and those that only participated in the initial census survey (Table 1). The mean age of participants was 21 years (95 % C.I. 20–22), 3 years less than the non-participants (24 years) (95 % C.I. 24–25) (*p* = 0.0001). The response among females (54 %) was higher than among males (46 %) in all age groups. The relative proportion of heads of the households to other family members was lower in the serosurvey participants compared to the non-participants. Participants were also more likely to be housewives and students, while manual and office workers were less likely to participate. Among subjects who were above the legal age of marriage in Egypt, participants were more likely to be married compared to non-participants. A lower proportion of participants were Manshiet Nasser natives and with a lower and moderate standard of living than non-participants.

**Table 1:** Demographic characteristic differences between participants (submitted a blood sample) and non-participants (only completed census survey) in HCV serosurvey in Manshiet Nasser, 2003

Socio demographic characteristics^a^	Participants *n* = 2180	Non-Participants *n* = 1576	*p*-value^*^
Mean Age (95 % CI)	21 (20–22)	24 (24–25)	0.0001
Head of House Hold	372 (17)	411 (26)	0.0001
Ever-Married	841 (65)	679 (60)	0.003
Education			
No education	616 (35)	442 (32)	0.05
Primary and Preparatory	831 (47)	555 (40)	<0.0001
Secondary and above	331 (18)	378 (27)	<0.0001
Current work status			
Not working	265 (15)	201 (15)	0.9
House wife/Students	965 (55)	545 (42)	<0.0001
Garbage collector, sorter and recycler	260 (15)	166 (13)	0.08
Manual laborer/drivers/shop workers	233 (13)	332 (25)	<0.0001
Office work	27 (2)	69 (5)	<0.0001
Origination			
Upper Egypt (Rural)	436 (20)	279 (18)	0.1
Lower Egypt (Rural)	24 (1)	26 (2)	0.01
Urban Governorates	401 (18)	460 (29)	<0.0001
Born in Mokkattam	1313 (60)	811 (51)	<0.0001
Standard of living considered to be poor	393 (18)	210 (13)	<0.0001

^*^Chi-square was used for n (%) while *T*-test was used for mean age

^a^All results are n (%) except for mean age (CI)

### Serosurvey results

**Fig. 2:**
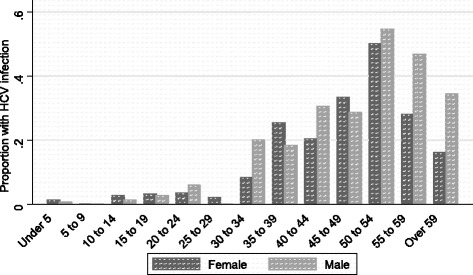
Prevalence (%) of HCV infection by gender and age group in Manshiet Nasser, 2003

A total of 180 subjects were found to have antibodies to HCV resulting in a crude overall prevalence of 8.3 % (Binomial exact 95 % C.I. 7.2–9.5 %). Among the serosurvey participants, the mean age for HCV positive subjects was 41 years (95 % C.I. 39–43) and only 19 years (95 % C.I. 18–20) for the HCV negative participants (*p* = 0.0001) (Table 2). Prevalence increased gradually with age with a sharp increase in the age group 31 years and above (PR = 54.6, *p* = <0.0001), a smaller peak at age 50 years, and a smooth decline thereafter (Fig. 2).

**Table 2:** Demographic characteristic differences between HCV-positive and HCV-negative volunteers in Manshiet Nasser, 2003

Demographic characteristics	HCV^a^				
	Positive	Negative	Crude Prevalence Ratio (PR)	Adjusted PR^c^	*P* Value
	*n* = 180^b^	*n* = 1989^b^			
	*n* = (%)	*n* = (%)			
Age (years)					
0–12	4/180 (2)	818/1989 (41)	1		
13–30	28/180 (16)	764/1989 (38)	7.0		<0.001
31 and above	148/180 (82)	407/1989 (21)	54.6		<0.0001
Mean^e^ (95 % CI)	41 (39, 43)	19 (18, 20)			
Male	89/180 (49)	903/1989 (45)	1.2	1.2 (0.9–1.5)	NS
Head of House Hold	89/180 (49)	282/1989 (14)	4.8	1.3 (1.0–1.7)	<0.05
Ever-Married^d^	155/172 (90)	685/1112 (62)	5.3	1.1 (0.6–2.1)	NS
Respondents with no education^e^	109/176 (62)	506/1599 (32)	3.2	1.4 (1.1–1.9)	<0.05
Current work status					
Not working/House wife/Students	77/161 (48)	1151/1587 (73)	1	1	
Garbage collector, sorter and recycler	40/161 (25)	220/1587 (14)	2.4	1.5 (1.1–2.2)	<0.05
Manual laborer/drivers/shop workers	41/161 (25)	192/1587 (12)	2.8	1.3 (0.9–1.8)	NS
Office work	3/161 (2)	24/1587 (2)	1.8	0.7(0.3–1.9)	NS
Ever sort garbage	83/180 (46)	558/1989 (28)	2.1	1.7 (1.3–2.2)	<0.0001
Previous residence					
Upper Egypt	86/179 (48)	348/1984 (17)	7.7	1.4 (0.9–2.0)	NS
Lower Egypt	7/179 (4)	17/1984 (1)	10.9	1.7 (0.8–3.3)	NS
Urban Governorates	49/179 (27)	352/1984 (18)	4.8	0.8 (0.5–1.2)	NS
Manshiet Nasser Natives	37/179 (21)	1267/1984 (64)	1	1	
Standard of living below community level	42/180 (23)	348/1989 (18)	1.4	1.4 (1.0–1.9)	0.05

^a^All results are n (%) except for mean age (CI)

^b^Numbers may not add up to (n) due to missing answers/eligibility to the question

^c^Adjusted for age

^d^Ever married respondents in the age of marriage (13 +) were compared to never married respondents within the same age category

^e^Respondents greater than 6 years who have no education is compared to respondents greater than 6 years who have any education

^f^NS is not statistically significant

### Marital status, education and governorate of origin

A positive HCV status was more prevalent among heads of households (*p* < 0.05), but not among those who had ever been married (even after adjusting for age). Subjects without formal education were more likely to have been infected (*p* < 0.05). Among HCV antibody positive subjects, the crude percent of subjects coming originally from Upper (Southern) Egypt was higher than those coming from Lower (Northern) Egypt, urban governorates, or Manshiet Nasser. After adjustment for age, the prevalence ratio was double for Lower Egypt compared to the urban governorates natives.

### Occupational risk factors

As compared to non-working individuals, including housewives and students, the garbage collectors, sorters or recyclers, and those who had ever worked in relation to garbage at any time during their lives and manual laborers had the highest HCV seroprevalence (Table 2). Although garbage sorting as a profession was associated with HCV infection, none of the occupation-related risk behaviors (e.g. not wearing gloves while sorting garbage, coming across medical waste, or being ever stuck with a needle or a sharp object during sorting) was specifically associated with infection. After adjustment for age, garbage collectors, sorters, or recyclers were more likely to be seropositive compared to respondents who were currently not working (aPR 1.5, *p* < 0.05). In addition those who had ever sorted garbage were more likely to be seropositive than those who never sorted any garbage (aPR 1.7, *p* <0.0001).

### Medical risk factors

A history of a surgical procedure or having received blood were associated with HCV seropositivity (Table 3). Thirteen percent of the anti-HCV positive subjects had a history of blood transfusion in contrast to only 3 % of the negative subjects with an adjusted prevalence ratio (aPR) of 2.0 (*p* < 0.0001). Having had a tattoo was also significantly associated with anti-HCV positivity (*p* < 0.05). Self-reported procedures were further analyzed according to the place where they were performed. There was a trend toward dental work done in private clinics to be associated with anti-HCV positivity. Although circumcision was not generally associated with a greater risk of having HCV, the procedure was significantly associated with HCV when performed in places other than a hospital (*p* < 0.0001, data not shown).

**Table 3:** Differences in self-reported potential risk factors for HCV acquisition between HCV-positive and HCV-negative volunteers in Manshiet Nasser, 2003

Probable risk factors	Anti-HCV^*^			
	Positive	Negative	Crude P.R.	Adjusted^b^
	n(%)	n(%)		P.R. (95 % CI.)
	*n* = (180)^a^	*n* = (1989)^a^		
Previous surgery	122/180 (68)	966/1989 (49)	2.1	1.3 (1.0–1.7)
Any medical procedure	95/176 (54)	588/1972 (30)	2.6	1.1 (0.8–1.4)
Previous dental work	139/180 (77)	993/1987 (50)	3.2	0.9 (0.7–1.3)
Ever received blood transfusion	23/176 (13)	57/1981 (3)	3.9	2.0 (1.5–2.8)^*^
Have any tattoos	103/180 (57)	927/1989 (47)	1.5	1.4 (1.1–1.9)^**^
Share razor blades	10/89 (11)	75/904 (8)	1.3	0.7 (0.4–1.3)
Go to the barber	89/89 (100)	858/893 (96)	N/A^c^	N/A^c^
Ever have abortion/deliveries	80/82 (97)	410/512 (80)	8.9	3.6 (0.8–16.0)
Received injection >10 injections	130/179 (73)	847/1986 (43)	3.4	1.5 (1.1–2.0)
Reuse of needles for injections	9/170 (5)	42/1681 (2)	2.0	1.0 (0.5–1.7)
Have ear/body piercing	92/180 (51)	1063/1988 (53)	0.9	0.9 (0.7–1.1)
Ever diagnosed of having Schistosomiasis	39/177 (22)	109/1967 (6)	3.8	1.5 (1.1–2.1)^***^
Have a family member has HCV	32/179 (18)	384/1960 (19)	0.9	1.0 (0.7–1.4)
Individual at work known to have HCV	11/168 (7)	104/1821 (6)	1.1	1.0 (0.6–1.6)
Never sort garbage with gloves	79/83 (95)	534/558 (96)	0.9	0.8 (0.4–1.7)
Ever come across medical waste while sorting	74/83 (89)	489/558 (88)	1.2	0.8 (0.5–1.4)
Ever stuck by any sharp object while sorting	77/83 (93)	494/558 (89)	1.6	1.0 (0.5–2.1)

^*^ *p* = <.0001

^**^ *p* = <0.05

^***^ *p* = <0.01

^a^Numbers may not add up to (n) or (100 %) due to missing answers/eligibility to the question

^b^Adjusted for age

^c^Not applicable

Twenty-two percent of the HCV positive subjects reported a history of schistosomiasis infection, compared to 6 % for those who were HCV negative, with an aPR of 1.5 (*p* < 0.01). The number of subjects reporting the receipt of injections for the treatment of schistosomiasis was too small for analysis.

A positive trend was also shown with the frequent use of injections. Seventy-three per cent of the anti-HCV positive subjects had a history of receiving more than ten injections during their life time.

### Living conditions

Participants with lower standard of living relative to the community tended to be seropositive (*p* = 0.05) (Table 2). However, among the living conditions measured, only the use of traditional latrines or using pits for sanitation (*p* < 0.001 and *p* < 0.05, respectively) were found to be associated with seropositivity (Table 4). Using multivariate analysis, those using traditional latrines were 2.1 times more likely to have HCV (*p* < 0.01) (Table 5).

**Table 4:** Differences in toileting facilities and crowding between HCV-positive and HCV-negative volunteers in Manshiet Nasser, 2003

Probable risk factors	HCV^a^				
	Positive, n(%)	Negative n(%)	Crude P.R.	Adjusted PR (95 % CI)^b^	*P* value
	*n* = (180)	*n* = (1989)			
The floor is made of earth & sand	12/179 (7)	100/1984 (5)	1.4	1.0 (0.6–1.7)	
Traditional latrine	148/179 (83)	1384/1984 (70)	2.0	2.0 (1.4–2.9)	<0.001
Use covered pit/uncovered pit for sanitation^c^	63/179 (35)	537/1984 (27)	1.4	1.3 (1.0–1.9)	<0.05
More than three persons per bedroom	55/178 (31)	596/1978 (30)	1.0	1.2 (0.8–1.6)	
No hand washing facility	36/179 (20)	326/1984 (16)	1.3	1.2 (0.8–1.6)	

^a^Numbers may not add up to (n) or (100 %) due to missing answers/eligibility to the question

^b^Prevalence Ratio Adjusted for Age (95 % Confidence Interval)

^c^Compared to municipal

**Table 5:** Multivariate analysis of demographic characteristics and risk behavior associated with HCV infection, Manshiet Nasser 2003

Demographic and risk factors*	PR (95 % CI)^a^	*p* Value
Age 13–30	14.4 (2.0–104.5)	<0.01
Age 31 and above	125.5 (17.2–915.1)	<0.0001
Head of Household	1.2 (0.8–1.7)	
No formal education	1.4 (0.9–2.0)	
Standard of living considered to be poor	1.2 (0.7–1.9)	
Ever Sort Garbage	1.5 (1.0–2.4)	0.05
Ever get blood transfusion	2.8 (1.5–5.2)	0.001
Have any tattoos	1.3 (0.8–2.0)	
Ever diagnosed with schistosomiasis	1.8 (1.1–2.9)	<0.05
Have a traditional latrine	2.1 (1.3–3.4)	<0.01

*All variables with *p* = 0.05 or lower were included in the multivariate model, the variable “Use covered pit/uncovered pit for sanitation” were dropped because it was affecting some of the other variables

^a^Prevalence Ratio (95 % Confidence Interval)

### Multivariate analysis

Results of multivariate analysis indicated that compared to the younger (<13 years) participants, those 13–30 years were 14.4 times more likely (*p* < 0.01) and those above 31 years were 125 times more likely to be HCV seropositive (*p* < 0.001). However, only the following risk factors were statistically significant: ever sorting garbage (*p* = 0.05), ever received a blood transfusion (*p* < 0.001), and ever diagnosed with schistosomiasis (*p* < 0.05) (Table 5).

## Discussion

### Regional HCV prevalence

The prevalence of HCV infection in Egypt, typically reported between 11 and 14 %, is among the highest in the world [23]. The Egypt Demographic Health Survey (DHS) 2008 estimated the prevalence at 14.7 % [10]. The seroprevalence in some of the neighboring eastern Mediterranean countries is much lower: Iran, 0.13 % [5]; Saudi Arabia, 0.4 to 1.7 % [8]; Syria, 0.95 % [9]; Pakistan, 4.7 % [7]; Libya, 1.2 %; Mauritania, 1.1 %; Algeria, 0.3 %; Morocco, 0.7 %; and, Tunisia 0.5 % [6]. The prevalence in other large developing countries such as China 0.9 % [24] and India 0.87 % [25] are also much lower than in Egypt. Therefore, major efforts are required to investigate all possible risk factors that may explain this unique prevalence, both the traditional well described risk factors reported by many authors and other less traditional potential risk factors that may be unique to this country. Although the prevalence rates in the DHS 2008 show a decline from previously reported higher rates, our study showed a trend to continuous transmission that should warrant more study of the risk factors for continuous transmission in the Egyptian population.

### HCV prevalence in Egypt

HCV infection varies significantly by region; an 8 % rate of infection was found in Cairo and Alexandria compared to 15 % in rural areas of the Nile Delta and Lower Egypt [26]. The age- and gender- adjusted prevalence rate of 9.1 % reported in this surveillance is slightly higher than those reported for the general Cairo population from previous reports [10]. This difference may be attributable, among other factors, to socio-demographic determinants and occupation of participants. The census conducted prior to the initiation of the surveillance activities and among households provided an accurate representation of the total area population as all households participated in the census survey. The demographic differences between participants and non-participants in the serosurvey as compared to the census were noted. These differences could have resulted from the fact that the blood sampling was conducted during times when men were expected to be at work. House to house “mop up visits” were conducted on days when this population usually does not work, but the differences in participation were not completely resolved. Additionally, the refusal rate was higher than estimated. Despite these differences, we feel the study results are generalizable because of the homogeneous nature of the population in Manshiet Nasser and the even distribution of the population in regards to the social and demographic characteristics within each cluster.

Heads of households, usually males, were significantly fewer among participants while younger children were substantially greater. A possible explanation for higher child participation is that parents in the sampled population were keener to have their children tested, while refraining from desiring to know their infection status. According to some housewives among the participants, their men either knew or suspected they were positive and did not want to be tested. We hoped that counseling of the HCV-positive subjects and the other participants of the study would help the community take precautions and measures to avoid spread of the disease and seek treatment.

### HCV prevalence in young age and evidence of ongoing transmission

The mean age for anti-HCV positive participants in our study was 41 years and there was a steady increase in prevalence of anti-HCV with age. These results are consistent with several other studies in urban and rural parts of Egypt [27–30]. The higher prevalence of anti-HCV in adults in our study might be attributed to iatrogenic transmission of the virus during historic schistosomiasis mass treatment campaigns in the 1970s [12, 26]. We report that the prevalence of HCV differed among age groups and indicate a possible cohort effect, supporting the observation that continuous transmission of the virus occurs in Egypt [31, 32]. Although the age group representing the highest prevalence rates of infection are adults, HCV is more common than would be expected in the 10 to 25 years age group and in children under the age of 5 (Fig. 2). Comparatively, Miller et al. found that the 2010 incidence of this disease in Egypt is continuing at a rate of ≈ 6.9/1,000 persons per year, indicating the possibility of continuous hyperepidemic transmission [32]. A relatively high seroprevalence in children may also indicate intrafamilial transmission of hepatitis C [33].

### The spread of HCV infection through medical settings

Multivariate regression analysis of demographic characteristics among participants indicated that age, history of schistosomiasis, garbage sorting, blood transfusions, and the use of traditional latrines or pits for sanitation are significantly associated with infection (Tables 4 and 5). Parenteral transmission through medical procedures, such as blood transfusion and frequent use of injections, is one of the key risk factors for the continuous transmission of HCV among the Egyptian population. This observation was noted by other authors [16, 34] and is supported by findings from other developing countries. A history of blood transfusion was reported in 26.1 % of anti HCV positive patients in a large study from China [24] and in another population-based study from India, where history of reuse of disposable syringes accounted for 80.76 % of HCV infections [25]. In Egypt, these exposures have been attributed to blood banks with inconsistent testing for HCV infection in the blood products before transfusion, the practice re-using needles to provide medical injections, and occupational exposure to accidental needle stick injuries [35]. These observations have led the Egyptian government and Ministry of Health to recognize the need to develop effective programs for monitoring and regulating the practice of invasive hospital procedures in the different levels of health care centers, with special emphasis on the primary and secondary health care centers in rural and underprivileged regions.

### Potential risk factors: garbage collection

The occupation of garbage collection and sorting has never been reported as a risk factor for HCV infection, but it certainly exposes individuals living in this community to refuse collected from areas throughout greater Cairo. Healthcare facilities, including private clinics and offices, may be a source of unsterilized biohazardous medical waste. The study by Talaat et al*.* [35] found that 93 % of healthcare workers in private healthcare facilities disposed of used needles in regular wastebaskets. Items such as needles, razor blades, and other discarded medical equipment may transmit the virus to people handling the waste. In our study, significant risk factors related to anti-HCV prevalence included the occupation of garbage collection and sorting. Although the lack of use of gloves as personal protective equipment during sorting was not found to be significantly associated with HCV infection in this study, the number of individuals who ever used gloves in this category may have been too low to make a comparable control group.

### Potential risk factors: use of traditional latrines or pits

Among the other identified risk factors, the use of traditional latrines and pits for sanitation was similar to findings for Hepatitis B virus infection risks from a serosurvey conducted in Pakistan [36]. Since HCV is not known to be transmitted by fecal exposure, this observation may represent a surrogate for some other exposure. It is consistent with the finding of risk among the lower socioeconomic groups in this community.

The current surveillance study used a 4^th^ generation anti-HCV EIA kit for the detection of HCV antibodies. This method was used to demonstrate previous infection with HCV rather than the number of active cases such as in the DHS 2008 study which used both methods for Public Health Policy planning [10].

## Conclusion

The results of this study affirm the public health importance of stopping transmission in urban as well as rural areas. The noteworthy finding that garbage collection is a risk factor for HCV transmission adds to the general understanding of the epidemiology of this disease. The identification of high risk target populations and activities is needed as part of the efforts to decrease transmission in Egypt. While HCV prevalence after the age of 30 could be attributed to anti-schistosomiasis injections, the prevalence in younger age indicates ongoing transmission. Our data seem to show that “vertical” transmission is not the key driver to continued elevated HCV prevalence in this population. Other factors are contributing to the ongoing transmission throughout life. These need to be clarified further as they provide an opportunity to intercede and decrease transmission.

The defined population represented by the Manshiet Nasser area of Cairo exists as a robust model for studying incidence and epidemiology of infectious diseases and is an invaluable population for future research. Given the large reservoir of HCV infection in the population, further effort needs to be made to identify and mitigate new infections.

### Future questions

As highly effective but very expensive therapy becomes available, decision models for the most effective ways to interrupt the cycle of transmission need to be created. Further clarification of transmission risks among vulnerable populations is key to the creation of those decision matrixes.
